# Positive Behavior Management: Assessment of Rugby Referees in Children Sport

**DOI:** 10.3390/ijerph182010949

**Published:** 2021-10-18

**Authors:** Katarzyna Płoszaj, Wiesław Firek, Paweł Gąsior, Ewa Malchrowicz-Mośko

**Affiliations:** 1 Faculty of Physical Education, Józef Piłsudski University of Physical Education in Warsaw, 00-968 Warsaw, Poland; katarzyna.ploszaj@awf.edu.pl; 2 Department of Physical Education and Sport, Podhale State College of Applied Sciences in Nowy Targ, ul. Kokoszków 71, 34-400 Nowy Targ, Poland; pawel.gasior@ppuz.edu.pl; 3 Faculty of Physical Culture Sciences, Eugeniusz Piasecki University of Physical Education in Poznań, 61-871 Poznań, Poland; malchrowicz@awf.poznan.pl

**Keywords:** referee, sport official, rugby, children sport, educational practice, pedagogical function

## Abstract

During children’s sports competitions, the referees play a special role. The referees are expected to be able to identify behavioral problems (of players, coaches, and fans), applying specific techniques to prevent negative behavior of players. Adapting these actions to the specifics of the group or individuals is crucial in providing a safe educational environment that promotes child development. The main objective of this research was to assess the quality of referees’ interactions with players in terms of *positive behavior management* and *proficiency* during rugby matches of children aged 6–12 years. Twenty-three rugby referees officiating matches organized by the Polish Rugby Union in Poland participated in the study. The research used the Referee–Players’ Interaction Assessment Scoring System tool. Additionally, referee–player interactions were recorded with a GoPro 8 camera along with audio from a wireless intercom. The significance of differences between the ratings for each indicator was tested by chi-squared test, while a non-parametric Wilcoxon signed-rank test was used to compare the mean ratings of *positive behavior management* and *proficiency*. The Mann–Whitney U-test was used to compare differences between assessments of experienced and inexperienced referees. The observations showed that referees were rated significantly higher in the *proficiency* dimension than in *positive behavior management* dimension. Nevertheless, both ratings represent an average level of quality of interactions with the players. The referee’s experience did not determine the quality of his or her interactions with the players in the specific dimensions. The following conclusion was drawn from the research: referees should be trained in the methods and techniques for managing player behavior and should act to prevent the occurrence of negative behavior, by presenting clear and understandable expectations to players before the match and using preventive measures.

## 1. Introduction

In the sport of children and young people, it is too simplistic to believe that sport automatically generates educational benefits for them. Jones [1] pointed out that moral values of sports competition are “taught not caught”. According to this scholar, moral education should result from a deliberate educational strategy (“taught”). If one adopts the “caught” philosophy, they must be aware that inappropriate behaviors can just as easily be adopted by players. Coaches, instructors, and physical education teachers too often tend to accept unsportsmanlike behavior if it leads to a win [2]. There is a tendency to blur the distinction between sport for fun and multilateral development of children and competitive sport. This manifests itself in teaching players ways to break the rules of the game, sharp practice in the field, arguing with referees, and faking fouls and injuries.

A young player should be a subject to which conscious and purposeful actions are taken, aimed at his or her multilateral (emotional, social, cognitive, moral, health-related) development. To date, numerous studies have discussed the problem related to the coach-pedagogue [3,4,5,6,7]. The role of parents in the sports environment has also been analyzed [8,9,10,11,12,13]. It was pointed out that these entities are important for educational practice in sport. When children compete in team sports, there is also a referee on the field, who is usually overlooked when considering the educational potential of sport [14]. Referees who are in direct contact with the players are treated as participants in the game because their decisions influence the players’ behavior and the outcome of the game itself [15]. It is emphasized that the way decisions are communicated and explained, and the skills involved in managing behavior, are not irrelevant to the match climate and experiences of young players [16,17].

Referees perform various functions on the playing field, including, among others, an educational function [14,18,19], which can be defined as the intentional transfer of knowledge and skills as well as ideals and patterns of conduct characteristic of sports culture, expressed primarily in the quality of interaction with the players, aiming to build a positive climate of the sporting event and organize it in such a way that it is a source of positive cognitive, emotional, social, and health experiences for the child. The referee performs this function as part of common educational interventions, whereas the other persons involved are coaches and parents [19]. This function does not apply (or is limited) to adult sports.

Previous research on the role of the referee in children and youth sport has been related to the referees’ ability to use techniques to shape prosocial behavior [20], their role as educators [18], and perspective on the educational practice of competitive youth games [14,21]. Płoszaj, Firek, and Czechowski [22] developed the R-PIASS tool to assess the educational function of a referee. The proposed normative model of the referee-educator assumes that interactions are the primary mechanism of his or her educational function. The educational function of the referee consists of three domains: emotional support, instructional support, and game organization. Our research focuses on the game organization domain, which consists of two dimensions: *positive behavior management* and *proficiency*.

During children’s sports competitions, the referee plays a special role. Plessner and MacMahon [23] emphasized that refereeing means not just mechanical decision-making but also problem-solving. The referee’s skills in terms of the identification of behavioral problems of players, applying specific techniques to prevent negative behavior of players, and adapting these actions to the specifics of the group or individuals are crucial in providing a safe educational environment that promotes child development [14,24,25]. Referees acknowledge that specific knowledge is required to effectively control behavior [26]. Research results indicate that reading the emotional states of players and predicting their actions is one of the preconditions for good refereeing [27,28,29]. Therefore, researchers emphasize that referees should take care of instilling appropriate norms in players, both through their own attitudes [14,30] and through the use of various types of prosocial behavior techniques [20,25]. For the purposes of the research, it is assumed that the *positive behavior management* dimension is related to the actions taken by the referee before the sporting event (checking the facilities, greeting the players and coaches), during the event (refereeing), and after the event (saying goodbye to the players, writing match reports). Referees should prevent negative behavior and redirect this behavior to a socially acceptable one. Furthermore, *proficiency* is the ability of the referee to organize the game smoothly and without unnecessary interruptions. All players should know what they are expected to do. One of the indicators of *proficiency* is the proper preparation of the referee for the sporting event in terms of his or her knowledge and the necessary refereeing equipment [22]. Research demonstrated that if the referee has an appropriate referee uniform, the players treat him or her with more respect and give the referee more authority [31,32].

Different sports require different competencies from referees. Plessner and MacMahon [23] distinguished three types of referees based on the number of players and physical and mental strain: interactors, sports monitors, and reactors. Our focus is on interactor referees in rugby “with high interaction and physical movement demands and often a large number of cues to process” [33] (p. 9). The term “interactors” implies a more developed communication component [34]. A rugby referee, who makes many decisions during one match, has many opportunities for educational interactions, both those related to the formation of social and moral attitudes based on the values of sport and to meet the cognitive and emotional needs of young athletes. In this context, it makes no sense to examine the “monitors” and “reactors”. Their educational opportunities are limited.

The main objective of this research is to assess the quality of referees’ interactions with the players during rugby matches of children aged 6–12 in terms of *positive behavior management* and *proficiency*. The aim of this study was also to examine whether refereeing experience determines the assessment of the quality of referee–player interactions.

The research was exploratory, which means that so far, the positive management of player behavior as part of the educational function of the referee is a relatively new and less explored research area [35]. Few quantitative studies have measured this phenomenon in children and youth sport. Dosseville, Laborde, and Bernier [36] argued that the interpersonal dimension of the referee–player relationship remains largely unexplored. This is important since the lack of referees’ knowledge and skills in this field may contribute to children leaving sport prematurely [37,38,39,40]. Arthur-Banning et al. [20] showed that players evinced more pro-social behavior on the sports field when referees were trained in the use of behavior management techniques. So far, sports referees have been studied for their educational function in football and handball [19]. The evaluation of the referees’ ability to manage the players’ behavior showed that they could improve in this aspect. Similar results were obtained in the *proficiency* dimension. It is worth checking how rugby referees are doing in this regard. The results of the study can be used by refereeing departments of sports associations during referee training to develop and introduce the dimension of behavior management into the system of evaluation and control of referees’ work, which is an important aspect of their educational function. In order to strengthen the postulate of training referees to support the educational practice in sport, theoretical considerations are not enough. Postulates must be founded on the results of empirical research.

## 2. Materials and Methods

### 2.1. Participants

The choice of sport and the selection of referees for the study was purposive. Our interest focused on the referees who, according to the typology of Plessner and MacMahon [23], interact with players in numerous ways and their behavior affects the course of the game. Twenty-three rugby referees officiating matches for players aged 6 to 12 years organized by the Polish Rugby Union in Poland participated in the study. Since only handful of people have referee licenses in Poland, almost all the people refereeing children and youth matches do not have such licenses and are persons appointed by the tournament organizer to act as referees. They are primarily coaches and current or former players without formal referee training. Only three of them had a referee’s license. The mean age of the referees was 25.2 years (SD = 8.78). The participants had a refereeing experience of 2.09 years (SD = 0.60). This is the practice followed by many Polish sports associations. It was arbitrarily assumed for the purposes of the study that an experienced referee has refereeing experience of more than one year (*n* = 8) and an inexperienced referee has an experience of one year or less (*n* = 15).

### 2.2. Measures

The Referee–Players’ Interaction Assessment Scoring System [4] was used to observe referees in terms of *positive behavior management* and *proficiency*. The R-PIASS tool was designed to assess the educational function of referees in children’s sport in three domains: *emotional support*, *instructional support,* and *game organization*. Since the purpose of the present study is to assess the effects of referees on players in terms of *positive behavior management* and *proficiency*, only the *game organization* domain of the R-PIASS tool was used. A detailed description of the two dimensions of the educational function of the referee and their indicators and the method of their evaluation is presented in Table 1, Table 2 and Table 3.

### 2.3. Procedures

The research was approved by the Senate’s Research Bioethics Commission of the Józef Piłsudski University of Physical Education in Warsaw, Poland (SKE 01–10–2020). As a second step, collaboration was established with the Polish Rugby Union to inform them about the intended research. The cooperation with the sports union was necessary because the union provided the dates of the competitions as well as a personal list of referees. Each referee was observed twice by the observer while refereeing a match for children aged 6–12. The observations were performed in May and June 2021. In order to assess the quality of the interaction, it was recommended to perform several 15–20 min observations [41]. Due to the nature of the sports competition, it was decided that the entire match should be observed. The observer watched and coded all the referee’s activities that took place on the sports field. Since the referee’s verbal communications were also assessed, the referee studied was also equipped with a wireless intercom (Ejeas Fbim). Additionally, referee–player interactions were recorded with a GoPro 8 camera (GoPro, Inc., San Mateo, USA) along with audio from a wireless intercom. The observer observed the referee immediately before, during, and after the match, taking notes in relation to the specific behaviors described in the R-PIASS tool (Table 1). Each match was followed by an analysis of the notes taken and the playback of the video for re-observation. Each referee’s behavior concerning *positive behavior management* and *proficiency* was rated using a three-point scale: poor (P), average (A), and good (G). The method of determining the rating of the *positive behavior management* and *proficiency* dimensions based on the evaluation of individual indicators is presented in Table 4. The referee’s final rating was the average of the two observations.

### 2.4. Analytic Strategy

Research data were analyzed using basic descriptive statistics such as means, medians, standard deviations, and percentages. The normality of the distribution of the variables was examined by the Shapiro–Wilk test and using skewness and kurtosis. The significance of differences between the ratings for each indicator was tested by chi-squared test, while a non-parametric Wilcoxon signed-rank test was used to compare the mean ratings of *positive behavior management* and *proficiency*. The Mann–Whitney U-test was used to compare differences between assessments of experienced and inexperienced referees. The level of significance was set at 0.05. The reliability of the R-PIASS tool was tested by Cronbach’s alpha coefficient. ICC estimates and their 95% confident intervals were calculated based on a single-measurement (k = 2), absolute-agreement, 2-way mixed-effects model. Calculations were performed using the PASW Statistic 18 (IBM Corp., Armonk, NY, USA).

## 3. Results

The referees’ management of the players’ behavior was assessed using three indicators: *expressing expectations*, *using preventative officiating,* and *redirecting negative behavior*. Each of these referee behaviors was rated on a three-point scale: poor, average, good. The detailed results of the observations are presented in Table 5. In the *expressing expectation* indicator, more than half of the referees (65.22%) were rated poor. One in five referees was rated highly in this aspect. The referees’ *preventing negative behavior* and *redirecting negative behavior* was rated significantly better (“good” by 65.22% and 60.87%, respectively).

Within the *proficiency* dimension three indicators were observed: *continuity and flow of a game*, *directing players*, and *referee preparation (knowledge and skills; referee equipment)*. The first two indicators were rated at a high level (good), with 86.96% and 78.26%, respectively. A cognitively interesting observation was made regarding *referee preparation (knowledge and skills; referee equipment)*. Two-thirds of the referees were rated average (65.22%).

Based on the ratings of the indicators, ratings of the *positive behavior management* and *proficiency* dimensions were evaluated on a scale of 1 to 7 points (with 1 indicating poor and 7 indicating good quality of the referee–player interactions). These results are presented in Table 6. The observations show that referees were rated significantly higher in the *proficiency* dimension than in *positive behavior management* dimension (Z = −2.780; *p* = 0.005). On a seven-point scale, both ratings represent an average level of quality of interactions with the players.

The statistical tests (Table 7) demonstrated that there were no significant differences in the ratings of the quality of referee–player interactions in any of the two dimensions tested *(positive behavior management*: Z = −0.099; p > 0.05; *proficiency*: Z = −1.328; *p* > 0.05). The reliability of the tool measured by Cronbach’s alpha is 0.730. The ICC _(behavior management)_ = 0.829 with 95% confident interval of an ICC estimate is 0.644–0.923. ICC for the *proficiency* dimension is 0.776 with 95% confident interval of an ICC estimate is 0.539–0.899. The level of reliability is “moderate” to “excellent”.

## 4. Discussion

The research assumes that the educational function of the referee is expressed, among other things, in appropriate game management [19], which consists of two dimensions: *positive behavior management* and *proficiency*. The main aim of the study was to assess the quality of referee–player interactions during children’s rugby matches in terms of these two dimensions. The aim of this study was also to examine whether referee experience determines the assessment of the quality of referee–player interactions. Refereeing experience did not affect the ratings in both dimensions. Therefore, all referees could be treated as one group. The results showed that referees in both assessed dimensions show an average level of quality of interactions with the players, thus indicating areas for improvement.

The sporting behavior, reflected in the respect for teammates, coaches, opponents, referees, and the game, does not occur by itself [42,43,44]. It must be taught and then practiced and enforced. Therefore, referees, like other professionals associated with youth sports, need to have adequate knowledge and skills [45,46]. The result of the study indicated that the quality of the referee’s interactions with rugby players in the *positive behavior management* dimension was rated as average (M = 4.48). A medium quality of behavior management means that the referee presents his or her expectations of the players’ behavior before or during the match, but the rules are difficult to understand or assimilate, or are not consistently enforced. The referee not only responds to events on the field but also tries to prevent behavioral problems from occurring, although sometimes he or she fails to do so or takes ineffective action. There are brief episodes of misbehavior during the game that affect a small number of players. Based on their research, Arthur-Banning, Paisley, and Wells [20] demonstrated that referees trained in the use of player behavior modeling techniques were able to elicit more frequent prosocial and sporting behaviors from young players compared to referees not trained in this area. Not only can a referee reinforce socially acceptable behavior and norms of conduct, but he or she can be a role model. This gives them a chance to trigger the ripple effect. The phenomenon means that a child who notices the referee helping a player up is likely to do the same in a similar situation, as he or she sees that this behavior is expected. If the child chooses to help and receives a verbal reward or another form of positive reinforcement, they are more likely to repeat the behavior. This attitude may be considered desirable by other team members as well. Consequently, the norm of helping during the games becomes something expected rather than unusual, thus creating a safer and more supportive environment for child development [47].

The referee’s expressing expectations about players’ behavior during a rugby match was rated as poor (65.22%). Most of the referees did not explain to the children what behaviors on the field are advisable and what should be avoided. Carson [48] argues that there is both positive and negative discipline. The referee’s pre-match setting of clear boundaries that should not be crossed [33,49] and, if this occurs, consistent response to behavior that does not conform to previous agreements [20], are examples of positive discipline that does not involve punishment (often equated with suffering, pain, loss, retaliation, or mistreatment) but consequences [48]. This approach is more beneficial for the psychophysical development of children and adolescents and for building positive attitudes towards physical activity [42].

Effective professionals are not reactive but anticipate problems before they occur and take preventive measures [42]. Proactive refereeing, similar to proactive coaching or teaching [50], involves taking into account individual and group needs, the learning environment, and the leadership style. Even in amateur sports, a referee can be well prepared for upcoming games. By asking other referees who have refereed the games of the specific teams before, or based on his or her experience, the referee can determine what kind of behavior patterns were manifested during the particular game. While this information may influence perceptions of certain players or team behaviors, it can also be a valuable way to diagnose (identify) emerging trends related to players’ behavior during the game [33]. Rugby referees were rated average in *using preventative officiating* (65.22%) and *redirecting negative behavior* (60.87%), indicating that they can also improve in these aspects. Mascarenhas et al. [25] argue that a referee should prevent negative behavior, and when such measures prove ineffective, they should redirect it into positive behavior using appropriate methods.

The quality of the referee–player interactions in the *proficiency* dimension was rated as average (M = 5.52). This means that referees can also improve the game organization to make it smooth and without unnecessary interruptions so that the players know what to do. This also concerns the referees’ knowledge, skills, and equipment. The results of the observation of referees officiating handball matches showed that in the *proficiency* dimension, they were also rated at the average level [51]. In contrast, soccer referees’ interactions with players in this dimension were rated as good [22]. Other studies have indicated that few referees use appropriate techniques to prevent interference during play [52].

The limitation of this research was that it was impossible to observe referees with Polish Rugby Union refereeing licenses because they do not referee child and youth matches. There would be no way to know if qualified referees would be assessed differently than amateur referees recruited from coaches, and current or former players. Limitations are also related to the structured observation method used. This method is useful for small-scale interaction studies. The typical problem of aligning the assessments of different observers did not occur in this study because all observations were made by a single observer. However, the phenomenon of a possible change in the natural behavior of the study participants related to their awareness that they are being observed (Hawthorne effect) cannot be avoided.

## 5. Conclusions

The literature on referees in children and youth sport often mentions behavior management or game management, which, among other things, affect the quality of the sporting event. In the case of children and youth sports, behavior management is considered an important part of the educational function of the referee. Good game management, without unnecessary interruptions and the occurrence of the negative behavior of young players, is not only aimed at a better perception of sports competition but, above all, at utilizing the educational potential of sport.

Sports competitions for children and young people should be, above all, a place for their multi-faceted development and gaining new experiences. This perspective indicates a great responsibility on the part of the referee and requires his or her knowledge and skills to positively control the behavior of young players. Therefore, it is important to consider competencies that help build positive interactions with players when developing a normative model of the referee. The referee–athlete relationship in children’s sports must change to a caring relationship. The referee must take responsibility for the care of the participants in the game, as must coaches, parents, and organizers. The support from a referee that is adequate for a specific period of a child’s life is critical to his or her social and emotional development. Therefore, the current approach of referees, focused solely on punishing players for breaking the rules of the game, needs to be changed and their ability to take positive and proactive action needs to be developed. The referee should prevent, reduce, and redirect negative behavior and promote socially acceptable one. Therefore, in game management, the referee has to take into account not only the spirit of the game but also social expectations about the educational potential of sports competition. The research made it possible to diagnose the current behavior of referees in this respect. The average ratings do not make them bad referees, but they indicate the areas in which they can improve. Concrete guidelines are formulated in the following conclusions: (a) referee training should be enhanced with contents from the field of developmental psychology and pedagogy, (b) referees should be trained in the methods and techniques for managing player behavior, and (c) referees on the pitch should act to prevent the occurrence of negative behavior, including by presenting clear and understandable expectations to players before the match and using preventive measures.

The results of the research may be used by the Referee Department of the Polish Rugby Union and other governing bodies of team sports in which the referee interacts with players in numerous ways. Furthermore, the findings can be useful for modifying referee training programs and in the evaluation of their work.

## Figures and Tables

**Table 1:** Criteria for the assessment of the quality of referee–player interaction in the dimensions of *positive behavior management* and *proficiency* [4].

**Positive Behavior Management**			
**Indicators**	**Poor (1,2)**	**Average (3,4,5)**	**Good (6,7)**
Expressing expectations	The referee does not present (before the match) his or her expectations regarding the players’ behavior during the game	The referee presents his or her expectations regarding the player behavior before the match, but not understandably or does not enforce them during the game	The referee presents clearly and understandably his or her expectations for players’ behavior before the game and enforces them during the game
Using preventative officiating	The referee does not attempt to prevent behavioral problems or does not notice the increasing negative climate.	The referee attempts to prevent behavioral problems on the pitch but does this not always or sometimes ineffectively.	The referee always attempts to prevent negative behavior and his or her actions are effective.
Redirecting negative behavior	The referee does not respond to the negative behavior of the players, and it continues over time	The referee responds to the negative behavior of the players, but his or her actions are not always effective, and the behavioral problems are extended over time.	The referee responds to the negative behavior of the players on an ongoing basis and his or her actions are effective, and the behavioral problems do not last.
**Proficiency**			
**Indicators**	**Poor (1,2)**	**Average (3,4,5)**	**Good (6,7)**
Continuity and flow of a game	The game is not smooth and there are unnecessary interruptions.	The game seems smooth, but there are sometimes unnecessary interruptions.	The game is smooth and there are no unnecessary interruptions.
Directing players	The game is not well organized, and the players often do not know what to do.	The game is well organized, but there are situations where players do not know what to do.	The game is well organized, and the players always know what to do.
Referee preparation - knowledge and skills - referee equipment	The referee is not prepared to referee the match, does not have the appropriate uniform and equipment, or there are often situations where he or she seeks consultation or browses game rules.	The referee is prepared to referee the match, but there are occasions when he/her consults or looks into the game rules or does not have all the referee’s equipment.	The referee is well prepared to referee the match, has the appropriate uniform and full refereeing equipment.

**Table 2:** Examples of low-quality and high-quality referee–player interactions in the dimension of *positive behavior management*.

Indicators	Poor	Good
Expressing expectations	At no point in the game did the referee express his expectations of desired behavior.	Immediately before the beginning of the match, the referee announced to the players on the pitch that on that day, he expected a player to apologize to his opponent after a foul by shaking their hands.
Using preventative officiating	A defender pushed an attacker away in the penalty area incorrectly on several occasions during one action.	During a break in play, the referee approaches the player and in a short and subtle conversation, informs him that the next such foul will lead to a more severe penalty.
Redirecting negative behavior	The referee is ignoring a situation where one player is insulting another, and this has been going on for a long time.	After a foul, the referee calls the fouler to apologize to the fouled person.

**Table 3:** Examples of low-quality and high-quality interactions in the dimension of *proficiency*.

Indicators	Poor	Good
Continuity and flow of a game	There are many overly long pauses in the game as the referee walks over to the other referee, asking who last touched the ball before it went out of play.	The game is smooth, and the referee is almost invisible on the field.
Directing players	After the referee’s whistle, no one puts the ball in play because no one knows why the play was stopped. Coaches yell to their players that there was a foul.	After the referee’s whistle, the players bring the ball into play without unnecessary delay, because everyone knows what the reasons and consequences of the interruption were.
Referee preparation - knowledge and skills - referee equipment	After a foul that should be punished by a red card, the referee communicates the decision to remove the player from the game verbally because he does not have a red card. The referee officiates the match in plain clothes. The referee blows the whistle to indicate the offense and the coaches scream that this rule no longer applies.	The referee waits until the players are ready to play before the game. The referee has an appropriate and neat referee uniform and all necessary equipment.

**Table 4:** The way to determine the assessment of *positive behavior management* and *proficiency*.

Three Indicators	Score	
P, P, P	1	POOR
P, P, A	2	
P, A, A	3	AVERAGE
A, A, A P, A, G	4	
A, A, G	5	
A, G, G	6	GOOD
G, G, G	7	

Abbreviations: P = Poor; A = Average; G = Good.

**Table 5:** Assessment of the quality of referee–player interactions in the dimensions of *positive behavior management* and *proficiency* during children’s rugby competitions (*n* = 23).

		Quality of Referee–Players’ Interactions			Chi-Squared Test *p*
Dimension	Indicators	Poor	Average	Good	
Positive behavior management	Expressing expectations	65.22%	13.04%	21.74%	0.005
	Using preventative officiating	13.04%	21.74%	65.22%	0.005
	Redirecting negative behavior	21.74%	17.39%	60.87%	0.019
Proficiency	Continuity and flow of a game	0.00%	13.04%	86.96%	<0.001
	Directing players	8.70%	13.04%	78.26%	<0.001
	Referee preparation - knowledge and skills - referee equipment	13.04%	65.22%	21.74%	0.005

**Table 6:** Descriptive statistics and normality assessment for the ratings of quality of referee–player interactions in *positive behavior management* and *proficiency* of rugby referees (*n* = 23).

Dimension	Mean (SD)	Median	Skewness	Kurtosis	Shapiro-Wilk *p*
Positive behavior management	4.48 (1.8)	5.0	−0.038	−0.907	0.089
Proficiency	5.52 (1.4) *	6.0	−1.442	1.696	<0.001

* Significantly (*p* < 0.05) higher than in the *positive behavior management* (Wilcoxon’s signed ranks test).

**Table 7:** Differences in the assessment of the quality of referee–players interactions in *positive behavior management* and *proficiency* dimensions between beginner and experienced referees (*n* = 23).

	Rugby Referees					
	Beginner (*n* = 15)		Experienced (*n* = 8)			
Dimensions					Mann Whitney U-Test	
	Median	$\overset{\text{¯}}{x}$	Median	$\overset{\text{¯}}{x}$	Z	p
Positive behavior management	4.00	11.90	5.00	12.19	−0.099	0.921
Proficiency	6.00	12.70	6.00	14.44	−1.328	0.184
